# Modelling of Elongational Flow of HDPE Melts by Hierarchical Multi-Mode Molecular Stress Function Model

**DOI:** 10.3390/polym13193217

**Published:** 2021-09-23

**Authors:** Leslie Poh, Esmaeil Narimissa, Manfred H. Wagner

**Affiliations:** 1 Department of Chemical Engineering, Technion—Israel Institute of Technology (IIT), Technion City, Haifa 32 000, Israel; leslie.poh@gtiit.edu.cn; 2 Department of Chemical Engineering, Guangdong Technion—Israel Institute of Technology (GTIIT), Shantou 515063, China; 3 Polymer Engineering/Polymer Physics, Berlin Institute of Technology (TU Berlin), Ernst-Reuter-Platz 1, 10587 Berlin, Germany; manfred.wagner@tu-berlin.de

**Keywords:** high density polyethylene, HMMSF model, viscoelastic flows

## Abstract

The transient elongational data set obtained by filament-stretching rheometry of four commercial high-density polyethylene (HDPE) melts with different molecular characteristics was reported by Morelly and Alvarez [Rheologica Acta 59, 797–807 (2020)]. We use the Hierarchical Multi-mode Molecular Stress Function (HMMSF) model of Narimissa and Wagner [Rheol. Acta 54, 779–791 (2015), and J. Rheology 60, 625–636 (2016)] for linear and long-chain branched (LCB) polymer melts to analyze the extensional rheological behavior of the four HDPEs with different polydispersity and long-chain branching content. Model predictions based solely on the linear-viscoelastic spectrum and a single nonlinear parameter, the dilution modulus $G_{D}$ for extensional flows reveals good agreement with elongational stress growth data. The relationship of dilution modulus $G_{D}$ to molecular characteristics (e.g., polydispersity index (PDI), long-chain branching index (LCBI), disengagement time $\tau_{d}$) of the high-density polyethylene melts are presented in this paper. A new measure of the maximum strain hardening factor (MSHF) is proposed, which allows separation of the effects of orientation and chain stretching.

## 1. Introduction

Polyethylene is the most broadly used polymer, with applications ranging from plastics packaging found in daily lives to engineering plastics. Processability of commercial thermoplastics has always been a key area of research as we continue to work towards more efficient and greener manufacturing. During polymer processing, polymers are subjected to shear and extensional flow [1,2] simultaneously with extensional flow playing a dominant role in processes such as blow molding [3], fiber spinning [4], film blowing [5] and film casting [6,7]. With processing behaviors of polymers being influenced by their molar mass (M_w_), polydispersity (PDI), and long-chain branching (LCB) [8], studying the effects of molecular characteristics and chain architecture on shear and extensional rheological behavior of polyethylene is important in optimizing its processability.

It is has been known that low concentrations of LCB can be beneficial to the processing characteristics of linear polyethylene [9]. However, conventional LCB detection methods that use size exclusion chromatography (SEC) and nuclear magnetic resonance (NMR) may face a challenge in detecting low levels of LCB [10,11,12]. Therefore, over the decades, various methods have been put forth to detect and determine low traces of LCB in linear polyethylene. Dilution rheology [13,14] can provide quantitative signs of LCB but requires the determination of the molecular weight distribution by SEC, and the method is restricted to long-chain branched polymers with well-defined topography, such as metallocene HDPE. The increase in the traces of LCB can be observed in the enhancement of zero-shear viscosity ($\eta_{0}$) [8,15]. However, due to the long relaxation times of branched polymers, it is not easy to get an accurate measurement of the zero-shear viscosity [16,17]. Moreover, the effects of molar mass, polydispersity, and LCB need to be separated, which is hard as the increasing contents of LCB and broadening of molecular weight distribution have similar effects on the shear rheological behavior [16,17,18]. The van Gurp-Palmen plot [19] has been used to determine molecular information of a given polymer in terms of its molecular weight, polydispersity and LCB [20,21,22,23]. Shroff and Mavridis [24] proposed a long chain branching index (LCBI) based on the ratio between the zero-shear viscosity and the intrinsic viscosity of linear polyethylene. Using similar principles, Garcia-Franco et al. [21] showed that there was a linear relationship between the LCBI to the loss angle  calculated from the van Gurp-Palman plots at complex modulus of $\left|G^{*}\right|=10\;\mathrm{kPa}$ for truly linear polyethylenes (hydrogenated anionically synthesized polybutadienes). Using the rule-of-thumb relation proposed by Garcia-Franco et al. [21], Morelly and Alvarez [23] proposed a simple qualitative index for LCB.

It is important to be able to directly relate the effects of molecular characteristics of a polymer to its processability, thus, to determine the extent of any improvement. This gap can be bridged by constitutive modeling of the shear and extensional rheological behaviors. A robust constitutive model capable of reaching quantitative agreement with the rheological properties of polymers can be applied in polymer processing simulation for testing the processability of polymers according to the processing conditions. This would also reduce the time and cost in physical testing of polymers to determine the suitable molecular structure of specific polymer processes and plastics applications. The Hierarchical Multi-mode Molecular Stress Function (HMMSF) model of Narimissa and Wagner [25,26,27,28,29] is based on the concepts of hierarchical relaxation and dynamic dilution, and takes into account the interchain tube pressure. The model accurately predicts the uniaxial and multiaxial extensional and shear rheological behaviors of linear and LCB polymer melts based on their linear-viscoelastic characterization. The HMMSF model features only a single non-linear material parameter, i.e., dilution modulus $G_{D}$, for extensional flows, and an additional constraint release (CR) parameter for shear flow. The HMMSF model was extended by Narimissa and Wagner [27,30] to encompass monodisperse, bidisperse, and polydisperse linear polymer melts by means of relating the relaxation times to the Rouse stretch-relaxation times for each relaxation mode, which makes the HMMSF model suitable for all commercial polymers. The HMMSF model is a great choice to be developed into numerical simulations of polymer processes due to its requiring of a maximum of two material parameters, its mathematical simplicity, and the quantitative prediction of rheological properties of polymeric melts [29,31]. Moreover, the HMMSF model is now available for linear and nonlinear rheological modeling in the IRIS software [32].

Morelly and Alvarez [23] studied four commercial grades of high density polyethylene (HDPE) with different molecular weight, polydispersity and level of LCB to analyze the effects that molecular architecture and distribution of molecular weight have on the extensional rheology of linear polyethylene. Morelly and Alvarez [23] concluded that while molecular weight and polydispersity affect the nonlinear rheological behavior of HDPE, even low traces of LCB have a significant influence on the extensional rheology. In the same study, Morelly and Alvarez proposed a simplified LCBI that is based on the loss angle (δ) calculated from the van Gurp-Palmen plot and a maximum strain hardening factor (MSHF), which was originally developed as the ratio between zero-shear viscosity of long-chain branched and perfectly linear polyethylene melts [33].

The objective of this paper is to systematically analyze the aforementioned commercial grades of HDPEs (investigated by Morelly and Alvarez [23]) by the HMMSF model. This study will attempt to relate these molecular characteristics with the dilution modulus $G_{D}$ of the HMMSF model. We also propose a new method to determine the extent of the strain hardening in polymers using molecular modeling.

## 2. Materials and Methods

### 2.1. Materials

Four high-density polyethylene (HDPE) samples, PE-191-8, PE-127-9, PE-124-10, and PE-236-12 were studied by Morelly and Alvarez [23] regarding the effects of polymer chain architecture and molar mass distribution on their elongational flow. The samples are named in the form of PE-A-B, where A denotes the molecular weight of the polyethylene (in kg/mol) and B denotes the polydispersity index (PDI). Details of the rheological measurements of the polymers can be found in Morelly and Alvarez. 

### 2.2. Modelling Approach

The Hierarchical Multi-mode Molecular Stress Function (HMMSF) model of Narimissa and Wagner for linear and long-chain branched (LCB) polymer melts implements the concepts of (i) hierarchical relaxation, (ii) dynamic dilution, (iii) interchain tube pressure, and (iv) convective constraint release [25,26,27,28,30]. The model consists of a well-defined set of constitutive relations based on clear and physically justified assumptions and comprises the rheology of both linear and LCB melts. 

The extra stress tensor of the Hierarchical Multi-mode MSF (HMMSF) model is given as,

(1) $$\sigma (t)=\sum_{i}\int_{-\infty }^{t}\frac{\partial G_{i}(t-t^{\prime })}{\partial t^{\prime }}f_{i}^{2}(t,t^{\prime })S_{DE}^{IA}(t,t^{\prime })dt^{\prime }$$

$S_{DE}^{IA}$ is the Doi and Edwards orientation tensor which is based on the assumption of independent alignment (IA) of tube segments [34], and which is five times the second order orientation tensor **S**,

(2) $$S_{DE}^{IA}(t,t^{\prime })\equiv 5\langle \frac{u^{\prime }u^{\prime }}{u^{\prime 2}}\rangle =5S(t,t^{\prime })$$

*u*′ represents the length of the deformed unit vector **u**′ at time *t*, and the bracket denotes an average over an isotropic distribution of unit vectors at time t′, **u**(t′), which can be expressed as a surface integral over the unit sphere. The molecular stress functions $f_{i}=f_{i}(t,t^{\prime })$ in Equation (1) are defined as the inverse of the relative tube diameters *a_i_* of each mode *i*,

(3) $$f_{i}(t,t^{\prime })=a_{i}{}_{0}/a_{i}(t,t^{\prime })$$

In the same way as the orientation tensor **S**, the molecular stress functions are functions of both the observation time *t* and the past time time *t*′, when tube segments are created by reptation. The relaxation modulus *G(t)* of the melt can be expressed as a sum of discrete Maxwell modes with partial relaxation moduli $g_{i}$ and relaxation times $\tau_{i}$,

(4) $$G(t)=\sum_{j=1}^{n}G_{i}(t)=\sum_{j=1}^{n}g_{j}\exp(-t/\tau_{j})$$

By considering the ratio of the relaxation modulus at time $t=\tau_{i}$ to the dilution modulus $G_{D}=G(t=\tau_{D})$, we determine the mass fractions $w_{i}$ of dynamically diluted linear or LCB polymer segments with relaxation time $\tau_{i}>\tau_{D}$,

(5) $$\begin{array}{l} w_{i}^{2}=\frac{G(t=\tau_{i})}{G_{D}}=\frac{1}{G_{D}}\sum_{j=1}^{n}g_{j}\exp(-\tau_{i}/\tau_{j})\;\;\;\;\;\;\;\mathrm{for}\;\;\;\tau_{i}>\tau_{D}\\ w_{i}^{2}=1\;\;\;\;\;\;\;\;\;\;\;\;\;\;\;\;\;\;\;\;\;\;\;\;\;\;\;\;\;\;\;\;\;\;\;\;\;\;\;\;\;\;\;\;\;\;\mathrm{for}\;\;\;\tau_{i}\leq \tau_{D} \end{array}$$

We assume that the value of $w_{i}$ obtained at $t=\tau_{i}$ can be attributed to the chain segments with relaxation time $\tau_{i}$, and we consider segments with $\tau_{i}<\tau_{D}$ to be permanently diluted, i.e., we fix their weight fractions at $w_{i}=1$. Although this may seem to be a very rough estimate, we could demonstrate that this is a sufficiently robust assumption to model the rheology of broadly distributed polymers, and that the modeling results are largely independent of the number of discrete Maxwell modes used to represent the relaxation modulus *G*(*t*) [25]. The evolution equation for the molecular stress functions $f_{i}=f_{i}(t,t^{\prime })$ of each mode can be expressed as [25],

(6) $$\frac{\partial f_{i}}{\partial t}=f_{i}(K:S)-\frac{1}{\alpha }(\frac{1}{\tau_{i}}+\beta CR)\;\left[(f_{i}-1)(1-\frac{2}{3}w_{i}^{2})+\frac{2}{9}f_{i}^{2}(f_{i}^{3}-1)w_{i}^{2}\right]$$

with the initial conditions $f_{i}(t=t^{\prime },t)=1$. The first term on the right hand side expresses affine deformation by stretch rate K:S with K being the velocity gradient tensor, the second term takes into account Rouse relaxation in the longitudinal direction of the tube, and the third term limits molecular stretch due to the interchain tube pressure in the lateral direction of a tube segment [35]. The topological parameter α depends on the chain architecture of the melt, and is given by,

(7) $$\begin{array}{l} \alpha =1\;\;\;\;\;\;\;\;\;\;\;\;\mathrm{for}\;\;\;\mathrm{LCB}\;\;\;\mathrm{Melts}\\ \alpha =1/3\;\;\;\;\;\;\;\;\;\mathrm{for}\;\;\;\mathrm{polydisperse}\;\;\;\mathrm{linear}\;\;\;\mathrm{melts} \end{array}$$

*CR* represents a dissipative Constraint Release (CR) term in shear flow which is zero in extensional flow. For more details about the HMMSF model, we refer to the review article [29]. 

The HMMSF model has also shown promising results in predicting the crystallization rate and morphology of HDPE [36] as well as extensional and shear rheology of low-viscosity polymer melts [37]. 

## 3. Comparison between Model Predictions and Data

**Figure 1:**
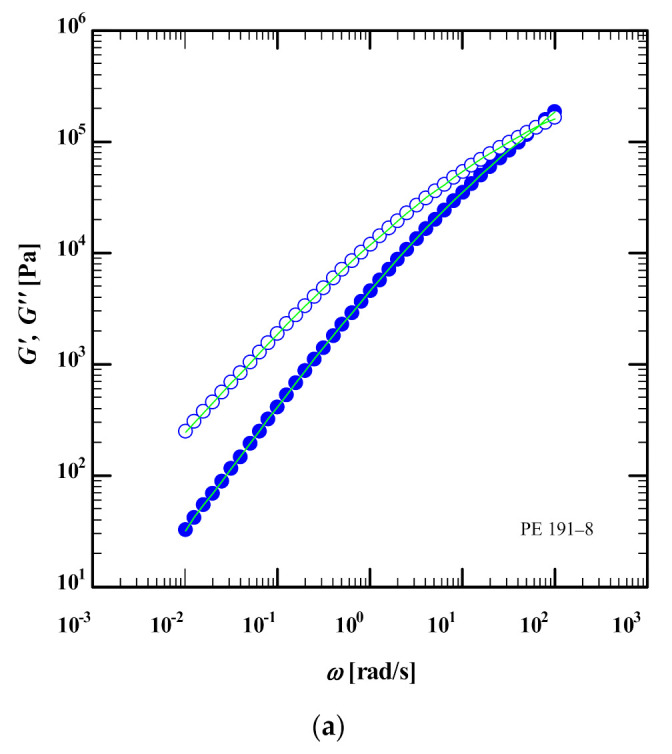

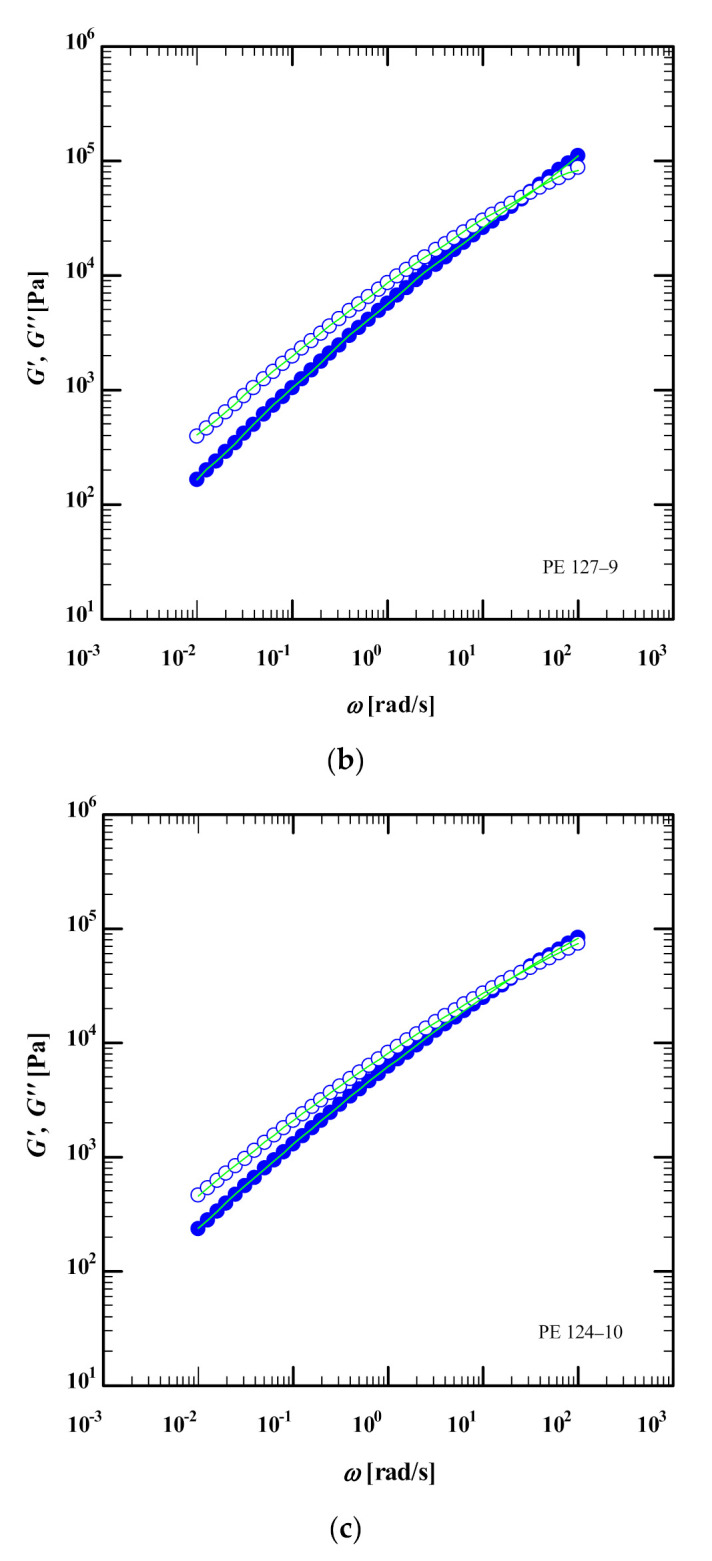

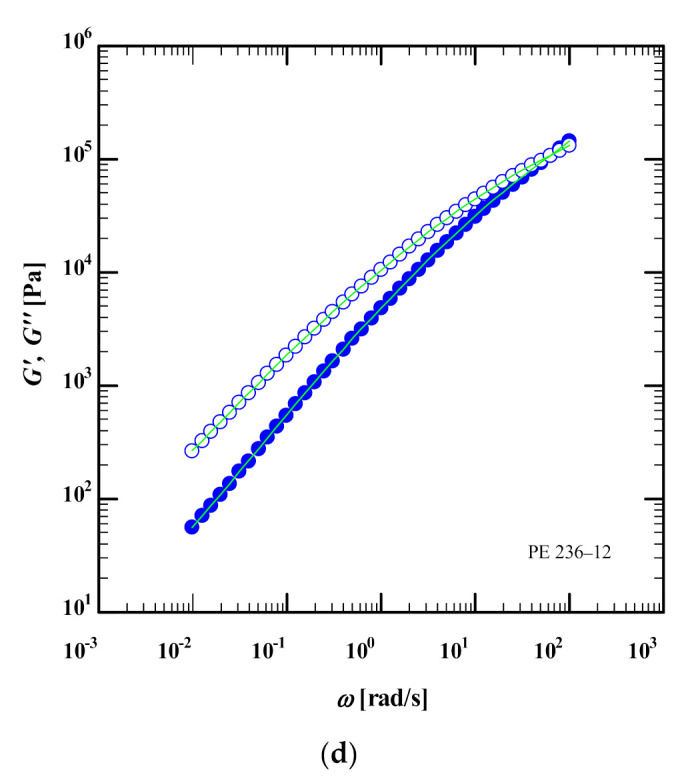
Storage (*G*′) and loss (*G*″) moduli of (**a**) PE 191-8, (**b**) PE 127-9, (**c**) PE 124-10 and (**d**) PE 236-12 at 160 °C (symbols) digitized from Morelly and Alvarez [23]. Continuous green lines represent fit by discrete relaxation spectrum (Equation (1) and Table 1) using the IRIS software [32].

All the shear and extensional data presented in this section are digitized from figures 1, 3b and 4 of the study by Morelly and Alvarez [23], and shall henceforth be referred to as M&A. The extraction of shear and extensional data was manually done using the WebPlotDigitizer [38] due to overlapping data points. The gaps seen in the presented data are due to obstruction from other data sets. The extracted extensional stress growth coefficient data are validated by comparisons between data digitized from figure 3b,c with figure 4 of [23]. The comparisons showed no change in data trend and strain hardening behavior, but a time offset was observed between the extracted data which will be further discussed in Section 3.2 of this paper.

### 3.1. Linear-Viscoelastic Characterization

**Figure 2:**
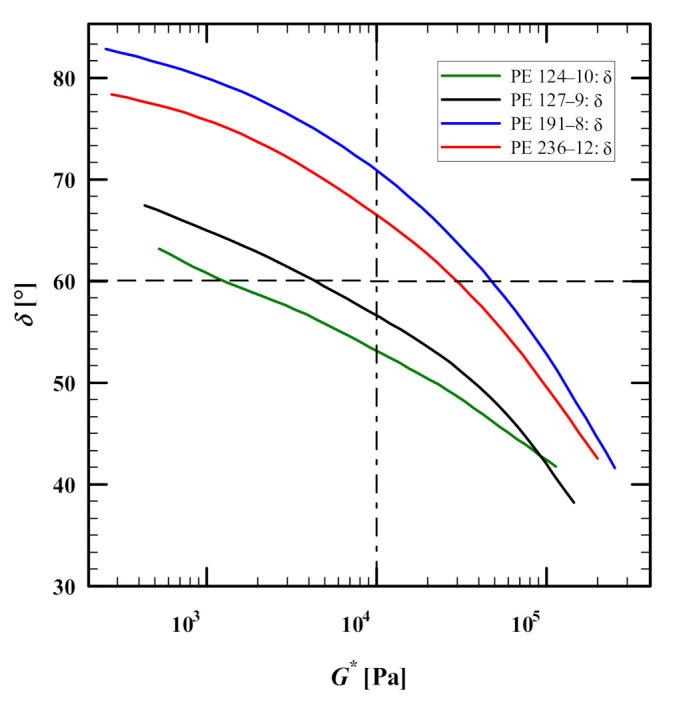
Van Gurp-Palmen plot for PE 191-8, PE 127-9, PE 124-10 and PE 236-12 at 160 °C produced in the IRIS software [32] using storage (*G*′) and loss (*G*″) moduli data from Morelly and Alvarez [23]. Horizontal (dashed) and vertical (dashed-dotted) lines denote the complex modulus at δ = 60 (for PDI), and loss angle at G* = 10 kPa (for LCBI, Equation (8)).

The linear viscoelastic data (storage *G*′ and loss modulus *G*″) of four HDPEs were digitized from figure 1 of [23]. Figure 1 shows the best fit (green lines) of *G*′ and *G*″ (symbols) of PE-191-8, PE-127-9, PE-124-10 and PE-236-12 at T = 160 °C using the IRIS software [32]. We used the IRIS software [32] to extract the relaxation spectrum from the data and to predict the linear viscoelastic behavior. From the relaxation spectrum, we also calculated the zero-shear viscosity $\eta_{0}=\sum_{i}g_{i}\tau_{i}$ of the HDPEs (Table 1), which is representative of the experimental frequency window, but we note that the true zero-shear viscosity may be significantly larger. We also note that the extracted relaxation spectrum using IRIS software has longer relaxation modes than the spectrum reported by M&A, and the underestimation of the weight of long relaxation modes in M&A results in poor prediction of the elongational viscosity at low strain rates [25]. From the linear viscoelastic data (storage modulus *G*′ and loss modulus *G*″), we used the IRIS software [32] to construct van Gurp-Palman plots (vGP), i.e., loss angle δ as a function of complex modulus G*, of the samples (Figure 2). The converging vGP plots show that neither polydispersity nor the molar mass plays a significant role in the rheological behaviours of the samples as δ→0 (or $G*\to G_{N}^{0}$). Bear in mind that as the HDPE samples are semi-crystalline, their plateau moduli cannot be easily determined. As shown by Trinckle and Friedrich [20], polydispersity has an inverse relationship with the magnitude of the complex viscosity at δ = 60° (chosen as somewhere between the terminal flow plateau, δ = 90°, and the minima of the curves). Here, this inverse relationship is evident for all HDPE samples except PE-236-12 (Figure 2). Furthermore, vGP curves can correlate the slope of G* to the molar mass of the polymer in the low loss angle region [20,23]. The long-chain branching index (LCBI) is another parameter that can be determined from the vGP curves at $G^{*}=10^{4}\mathrm{Pa}$ [21]. This will be explained in Section 4.1. 

### 3.2. Extensional Stress Growth Coefficient

**Figure 3:**
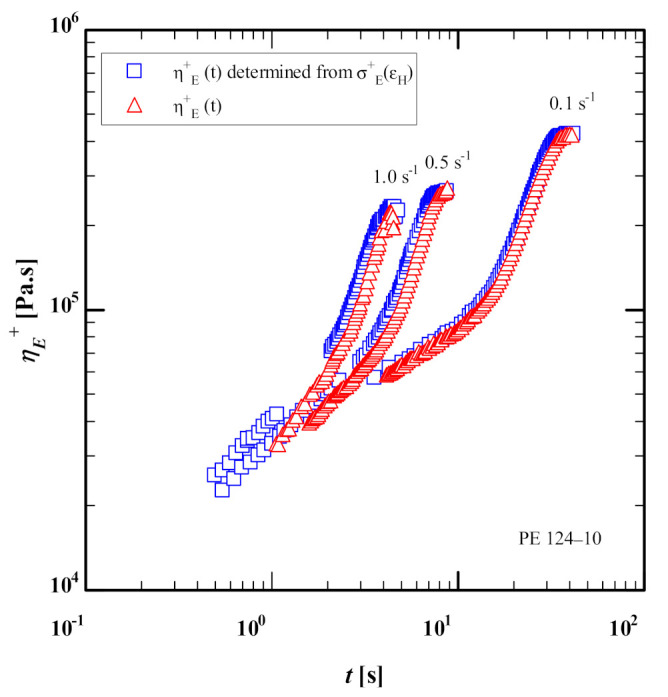
Comparison between extensional stress growth coefficient data as a function of time of PE 124-10 digitized from figure 3b (red triangle symbols) and figure 4 of [23] (blue square symbols) of Morelly and Alvarez [23].

Using the data of M&A, figure 3 of [23] shows discrepancies between the elongational stress growth coefficient as a function of time for PE 124-10 reported by M&A (figure 3b of [23]) on one side, and the stress growth coefficient calculated on the other side from the extensional stress as a function of Hencky strain (data from figure 4 of [23]) by dividing with the corresponding strain rate ($\dot{\varepsilon }=0.1,\;0.5,\;1/s$). The same discrepancy between stress growth coefficients reported and calculated from the elongational stress was also observed for the other HDPE investigated by M&A. The discrepancy between the elongation stress growth coefficients amounts to a time offset of approximately 0.3 s (Figure 3). The same time offset of 0.3 s was observed in the data of the other three samples reported by M&A and measured by a filament-stretching rheometer (VADER1000). It is important to note that a similar time offset has also been observed in the filament-stretching rheometer (VADER1000) in our lab. Therefore, as a result of this time offset, all the elongational stress growth coefficient data presented in this paper will be determined from M&A’s extensional stress as a function of Hencky strain (figure 4 of [23]).

**Figure 4:**
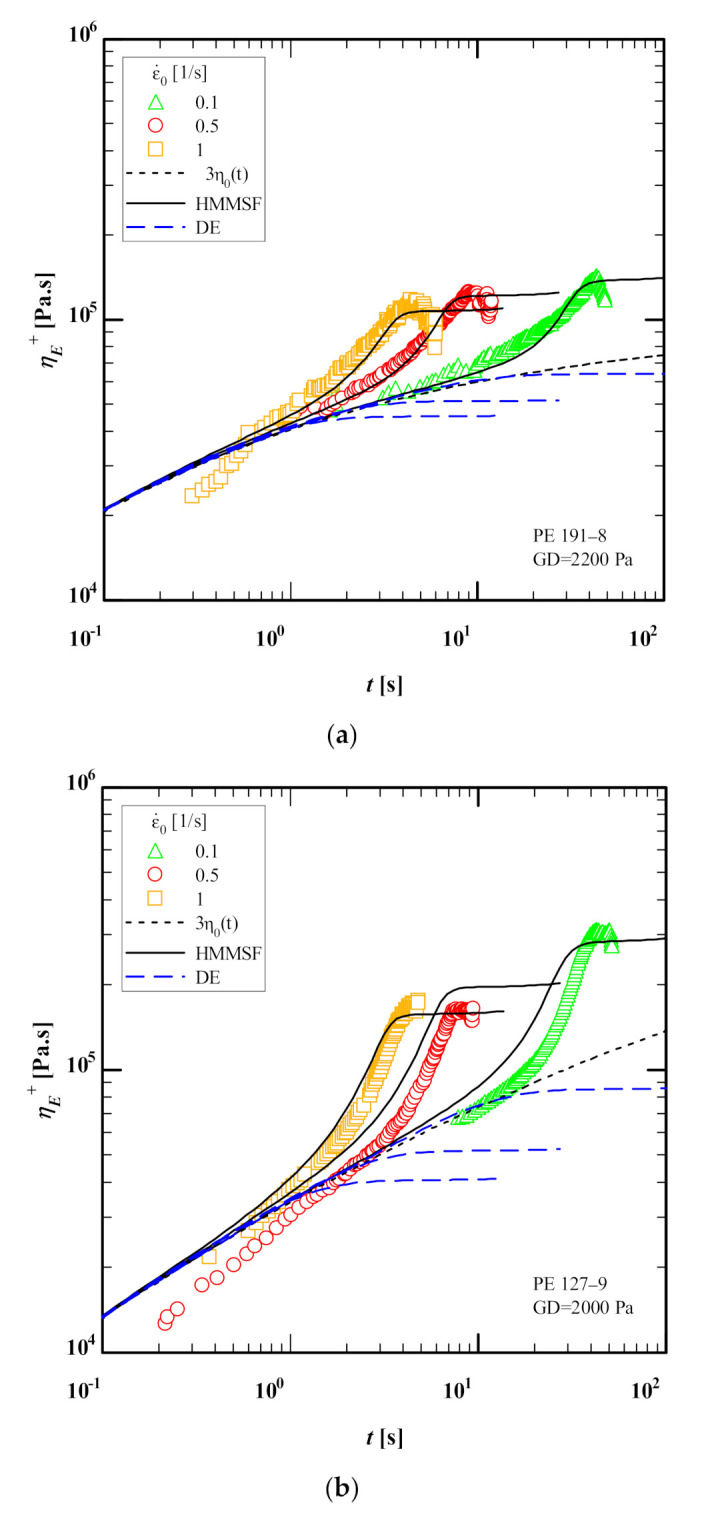

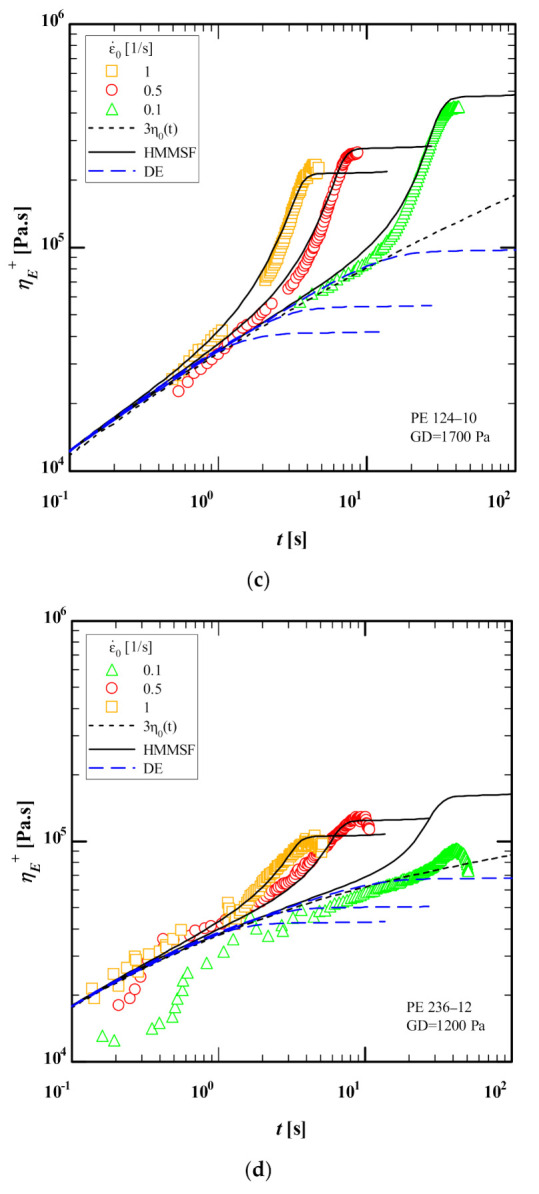
Comparison between data (symbols) of the extensional stress growth coefficient as a function of time of (**a**) PE 191-8, (**b**) PE 127-9, (**c**) PE 124-10 and (**d**) PE 236-12 at 150 °C with predictions of the Doi-Edwards (DE) model (blue dash lines) and the HMMSF model (Equations (4)–(6)) (continuous black lines) using the IRIS software [32].

Figure 4 compares the elongational stress growth coefficient as a function of time of PE 191-8, PE 127-9, PE 124-10 and PE 236-12 at 160 °C with predictions of the Doi-Edwards (DE) model [39] and HMMSF model (Equations (4)–(6)) using the IRIS software with dilution modulus $G_{D}$ of 2200, 2000, 1700 and 1200 Pa for PE 191-8, PE 127-9, PE 124-10 and PE 236-12, respectively, and using the relaxation spectra given in Table 1. Despite the limited linear-viscoelastic frequency window (a single SAOS measurement at 160 °C), excellent agreement between HMMSF model predictions and experimental data is achieved for elongational stress growth coefficient of PE 191-8, PE 124-10 and PE 236-12, while the observed deviation for PE 236-12 at Hencky strain rate ($\dot{\varepsilon }=0.1/s$) is most likely due to a measurement issue as it sits below the linear-viscoelastic envelope (LVE) as compared to the other two Hencky strain rates ($\dot{\varepsilon }=0.5\;\mathrm{and}\;1/s$). We observe fair quantitative agreement between the HMMSF model predictions and the experimental data even at the lowest strain rate ($\dot{\varepsilon }=0.1/s$), which would be most affected by the underestimation of the weight of longest relaxation modes. Generally, it is important to extend the experimentally accessible frequency window by using time-temperature superposition of multiple SAOS measurements at higher temperatures and extract the relaxation spectra from the mastercurve. Nonetheless, the HMMSF model can provide satisfying quantitative agreement between predictions and experimental data despite the limited linear viscoelastic frequency window. The overshoots in several elongational stress growth coefficients seen in Figure 4 are most likely not true maxima and they are rather signs of inhomogeneous deformation which will be further discussed in Section 4.2.

**Figure 5:**
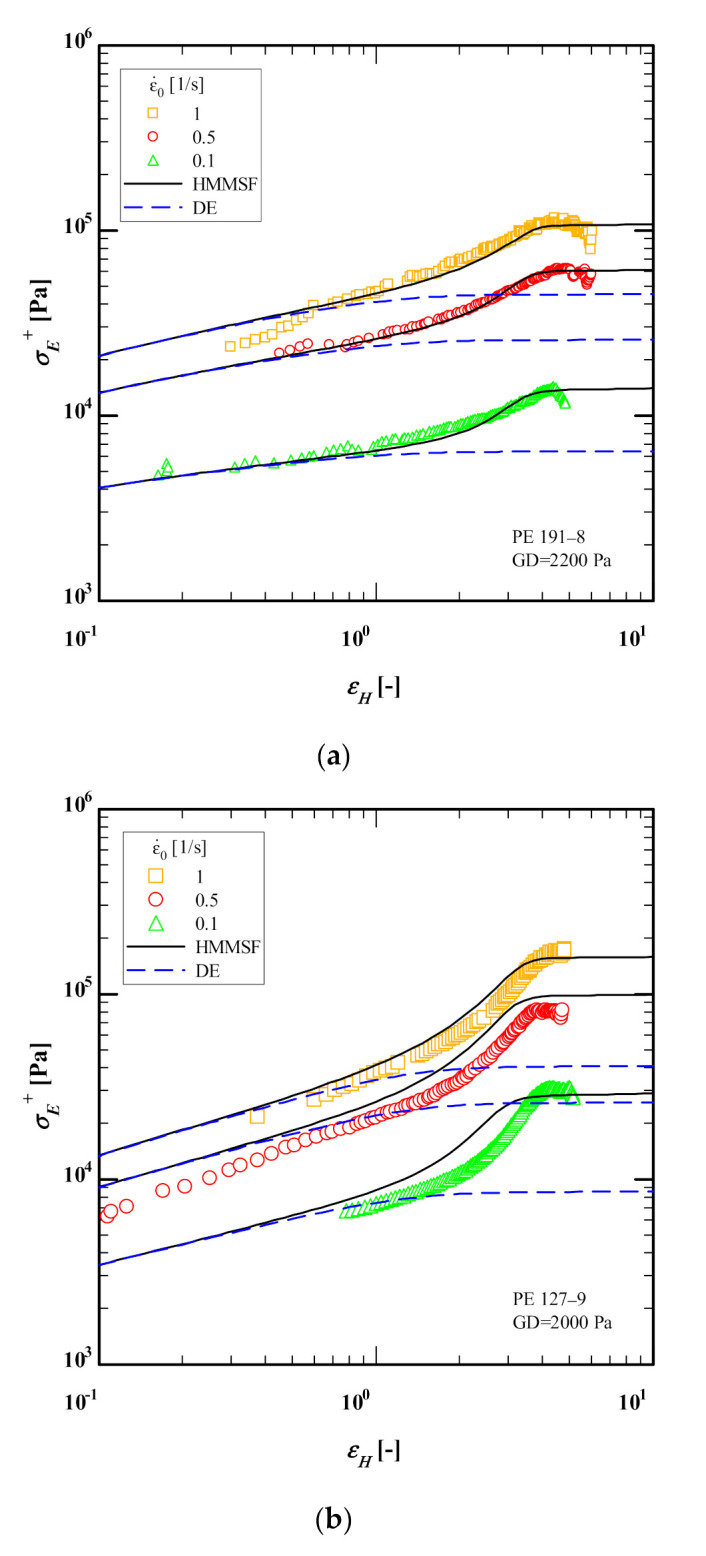

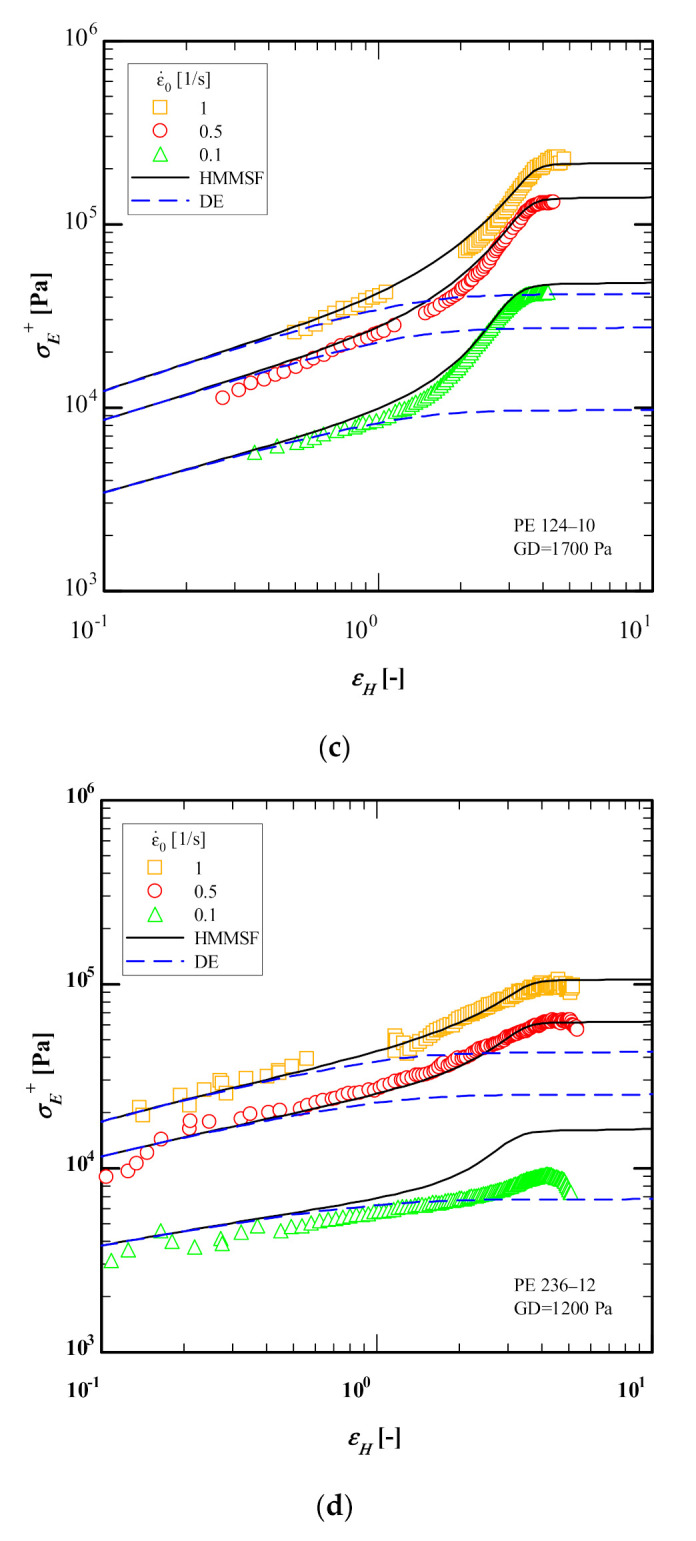
Comparison between data (symbols) of the extensional stress as a function of Hencky strain of (**a**) PE 191-8, (**b**) PE 127-9, (**c**) PE 124-10 and (**d**) PE 236-12 at 160 °C with predictions of the Doi-Edwards (DE) model (blue dash lines) and the HMMSF model (Equations (4)–(6)) (continuous black lines) using the IRIS software [32].

Figure 5 compares the extensional stress as a function of Hencky strain of the HDPE samples at 160 °C to the predictions of the Doi-Edwards (DE) model [39] and HMMSF model (Equations (4)–(6)) using again the IRIS software with dilution moduli $G_{D}$ of 2200, 2000, 1700 and 1200 Pa for PE 191-8, PE 127-9, PE 124-10 and PE 236-12, respectively, and using the relaxation spectra given in Table 1. Despite the mentioned limited linear-viscoelastic frequency window, excellent agreement between HMMSF model predictions and experimental data is achieved for the extensional stress of all four HDPEs. Once again, the observed deviation for PE 236-12 at Hencky strain rate ($\dot{\varepsilon }=0.1/s$) is most likely due to a measurement issue as it seems to fit closer to the tube model of Doi-Edwards which is for non-stretching monodisperse linear melts.

Overall, the predictions of the HMMSF model are in quantitative agreement with the experimental data of M&A despite the limited linear-viscoelastic frequency window.

## 4. Discussions of Polydispersity, Molecular Architecture, and Strain Hardening

### 4.1. Relationship between Dilution Modulus, Polydispersity, and Long Chain Branching Index

**Figure 6:**
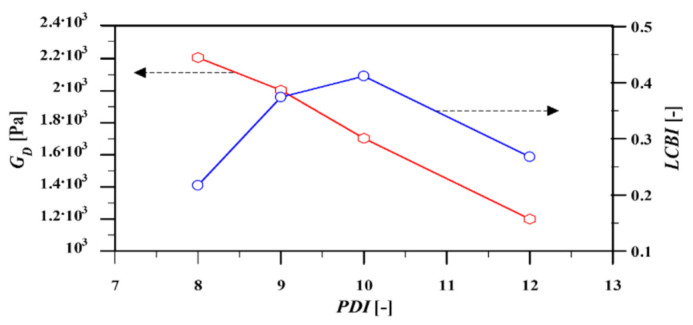
Relationship between dilution modulus ($G_{D}$, polydispersity index (PDI), and long chain branching index (LCBI).

The relationship between the dilution modulus of the HDPE samples obtained by fitting the single nonlinear parameter of the HMMSF model ($G_{D}$) to the experimental data, and the polydispersity index (PDI) of the samples is illustrated in Figure 6. It is evident that there is an inverse relation between PDI and $G_{D}$, meaning that increasing PDI leads to the increase in dynamic dilution, i.e., a decreasing dilution modulus. This is in line with the basic assumption of the HMMSF model: A broader molecular weight distribution with an increased concentration of short chains leads to enhanced permanent dilution, which in turn reduces the value of the dilution modulus.

M&A calculated the long chain branching index (LCBI) according to a rule-of-thumb based on the relation reported by Garcia-Franco et al. [21],

(8) $$LCBI=1-\delta (G^{*}=10^{4}\mathrm{Pa})/90{}^{\circ}$$

Figure 6 shows that except for the HDPE with PDI = 12 (i.e., PE-236-12), the LCBI of the samples increases as their PDI increase. This observation may indicate the dependence of long-chain branching (LCB) on the polydispersity. This will be investigated in more detail in the following parts.

The maximum strain hardening factor (MSHF), as used by M&A, follows the zero-shear viscosity enhancement factor of Stadler and Münstedt [33], and represents the ratio between the elongational stress growth factor $\sigma_{E}(t)$ of the polymer and the time-dependent linear-viscoelastic envelope of the stress growth factor $\eta_{LVE}(t)=3\eta_{0}(t)$,

(9) $$MSHF=\max\left(\frac{\eta_{E}^{+}(t,\dot{\varepsilon })}{\eta_{LVE}(t)}\right)$$

**Figure 7:**
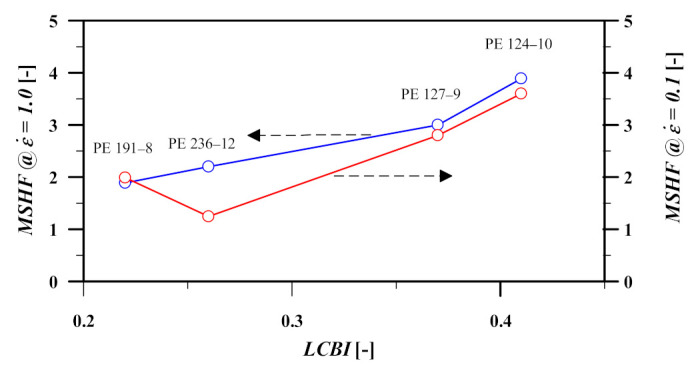
Relationship between MSHF and long chain branching index (LCBI). Data obtained by digitization of figure s3 of [23].

Using the values of figure s3 of [23], Figure 7 shows the relation between LCBI, MSHF, and PDI of the samples at strain rates of 0.1, and 1 s^−1^. Except for PE-236-12 @ $\dot{\varepsilon }=0.1/s$, a direct relationship is seen between MSHF and LCBI which emphasizes the role of traces of LCB in the strength of the strain hardening behavior of linear PE during elongational flow. The mentioned exception is in line with the erroneous stress growth coefficient data of the same sample reported in Figure 4 of Section 3.2.

The effect of LCBs, when there are only traces of LCBs and these are predominantly of a star shape, is represented in the LVE spectrum by broadening the spectrum, but this has no additional effect on the elongational viscosity (in addition to the effect that longer relaxation times lead to higher stretch). Therefore, it may be of interest to calculate the second moment of the relaxation spectrum, i.e., the disengagement (or longest) relaxation time $\tau_{d}$. The disengagement time ($\tau_{d}$), and the linear-viscoelastic zero-deformation viscosity in elongational flow ($\eta_{LVE}$) can be calculated from the relaxation spectrum as,

(10) $$\begin{array}{l} \tau_{d}=\frac{\sum_{i}g_{i}\tau_{i}^{2}}{\sum_{i}g_{i}\tau_{i}}\\ \eta_{LVE}=3\eta_{0}=3\sum_{i}g_{i}\tau_{i} \end{array}$$

**Figure 8:**
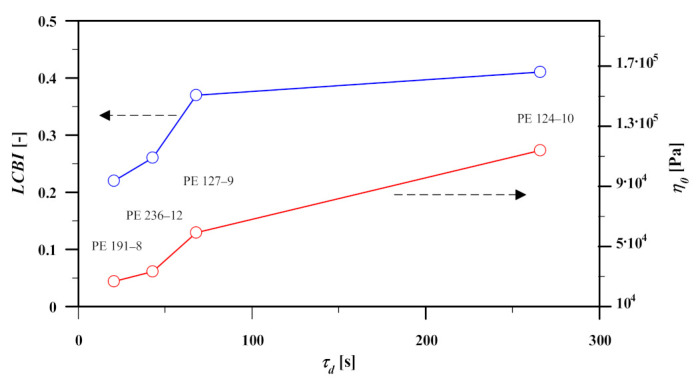
Relationship between PDI, LCBI (Equation (8)), and $\tau_{d}$ and $\eta_{0}$ (Equation (10)).

We note again that these quantities are based on the relaxation spectra obtained from SAOS measurements in the experimental window. Figure 8 shows the relationship between the PDI, LCBI (Equation (8)), and $\tau_{d}$ and $\eta_{0}$ (Equation (10)). There is a direct relation between all those rheological parameters (except the mentioned case for the PDI of PE-236-12). As both disengagement time and LCBI are related directly, we can confirm the validity of Equation (8) as estimation of the amount of LCB in linear melts. Henceforth, we will use the disengagement time as the measure of LCB.

As already mentioned, the minor maxima observed in the extensional data (Figure 4) are not true maxima and they are rather signs of inhomogeneous deformation (i.e., necking) at high strains as opposed to being a material property of HDPE (for more detail, see [40,41,42]). Using the HMMSF model, and considering the fact that the occurrence of stress overshoots (maxima) is unlikely in linear melt with only traces of LCB, we can define M&A’s MSHF (Equation (9)) as the ratio between the steady-state elongational viscosity and the LVE, $\eta_{E}(\dot{\varepsilon })/\eta_{LVE}$, as both values are readily accessible in the IRIS software (Figure 4). In order to determine the steady-state elongational viscosity and LVE, we can use the corresponding values of the stress growth coefficient and LVE at Hencky strain $\varepsilon_{H}=5$ which is experimentally accessible by filament stretching rheometers,

(11) $$MSHF_{HMMSF}=\frac{\eta_{E}^{+}(\dot{\varepsilon },\varepsilon_{H}=5)}{\eta_{LVE}(\varepsilon_{H}=5)}$$

**Figure 9:**
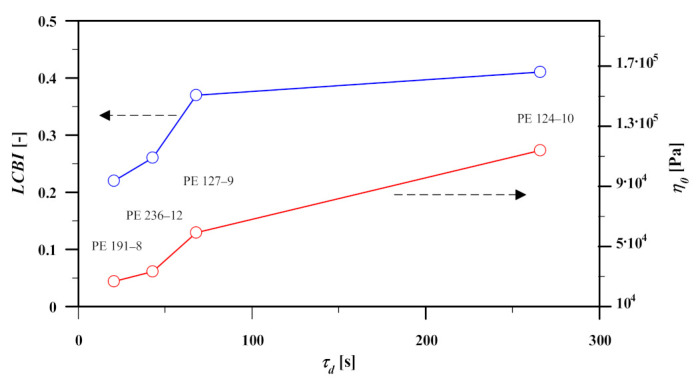
Comparison between the MSHF values of MA [23] (triangle, Equation (9)) and those generated by the HMMSF model (circle, Equation (11)).

Figure 9 shows the comparison between M&A’s MSHF (figure s3 of [23]) and those obtained from the HMMSF model, both as functions of the disengagement time and strain rate.

Ignoring the mentioned error (PE 236-12 @$\dot{\varepsilon }=0.1/s$), both methods show a direct relationship between LCB and the MSHF independent from the PDI. In contrast to M&A’s values, the HMMSF model (Equation (11)) shows that the MSHF is rather strain rate insensitive. The observed rate-insensitivity of the MSHF will be discussed in more detail next.

### 4.2. A New Approach for Evaluating Strain Hardening

We propose a new method for investigating the strength of the strain hardening behavior in polymers based on the molecular modelling. The tube model of Doi-Edwards [39], which was originally developed for monodisperse linear melts, assumes the tension in the deformed chain is equal to its equilibrium value even in the nonlinear viscoelastic regime, which is commensurate with the assumption that the tube diameter remains constant with deformation [31]. According to the model, chains are only oriented, but not stretched. Therefore, the segmental tube orientation $S_{\mathrm{DE}}$ is the only contributor to the extra-stress tensor **σ**, and if expressed as a single integral constitutive equation, it is given by,

(12) $$\sigma (t)=\int_{-\infty }^{t}\frac{\partial G(t-t^{\prime })}{\partial t^{\prime }}S_{\mathrm{DE}}(t^{\prime })dt^{\prime }$$

Therefore, by finding the ratio between the steady-state viscosities of the HMMSF model ($\eta_{E}(\dot{\varepsilon })$) and the DE model ($\eta_{DE}(\dot{\varepsilon })$) at a Hencky strain well-within the steady-state regime (e.g., $\varepsilon_{H}=5$ which is also experimentally accessible), we can define a more robust measure of strain hardening since the effects of orientation and stretch are distinctly separated. Hence, our proposed MSHF becomes,

(13) $$MSHF_{HMMSF/DE}=\frac{\eta_{E\_HMMSF}^{}(\dot{\varepsilon },\varepsilon_{H}=5)}{\eta_{E\_DE}^{}(\dot{\varepsilon },\varepsilon_{H}=5)}$$

As both models are readily available in the IRIS software, calculating the MSHF is an effortless task (see Figure 11d).

**Figure 10:**
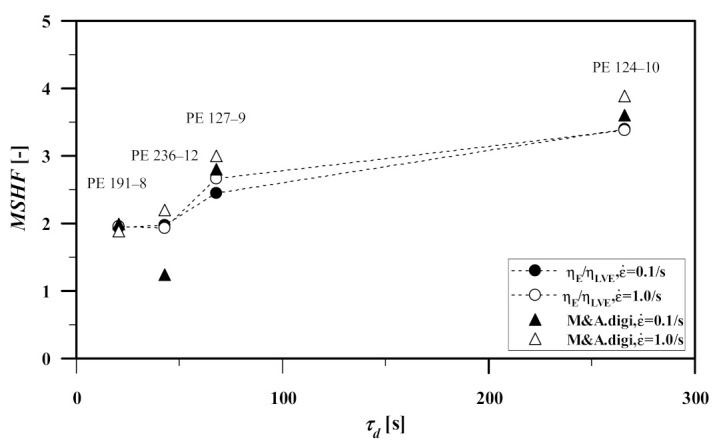
Proposed MSHF (Equation (13)) for PE samples at different strain rates.

Figure 10 demonstrates the values of our proposed MSHF (Equation (13)) at different rates. It is obvious that the MSHF is rather rate-insensitive for all three rates, and it becomes rate-independent for the 2 highest elongation rates investigated ($\dot{\varepsilon }=0.5,1/s$). The observed rate-insensitivity/independence indicates that the stretch part of the elongational flow becomes saturated in the experimental window (at $\varepsilon_{H}=5$). To examine this observation, one must investigate the spectral dependence of the stretch (see figure 3c of [25]).

**Figure 11:**
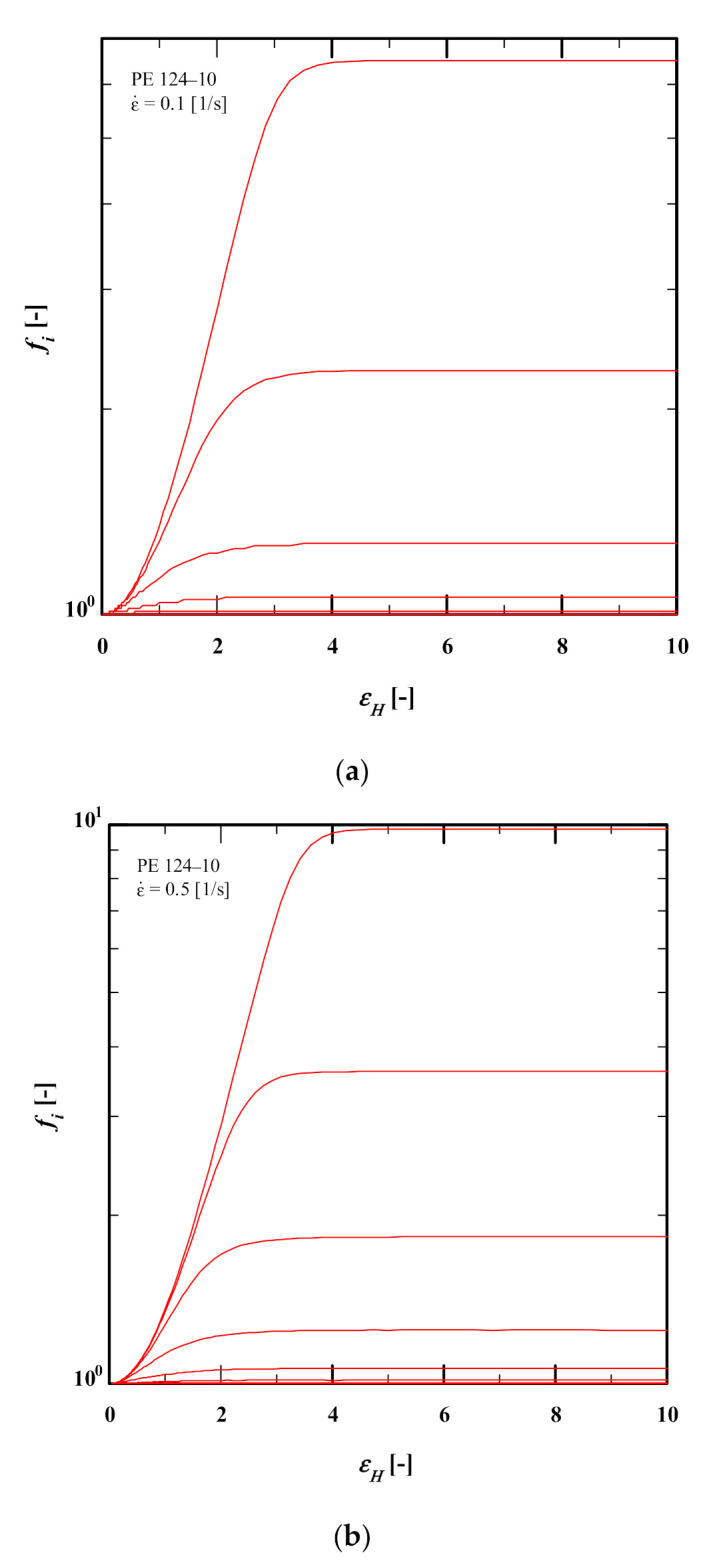

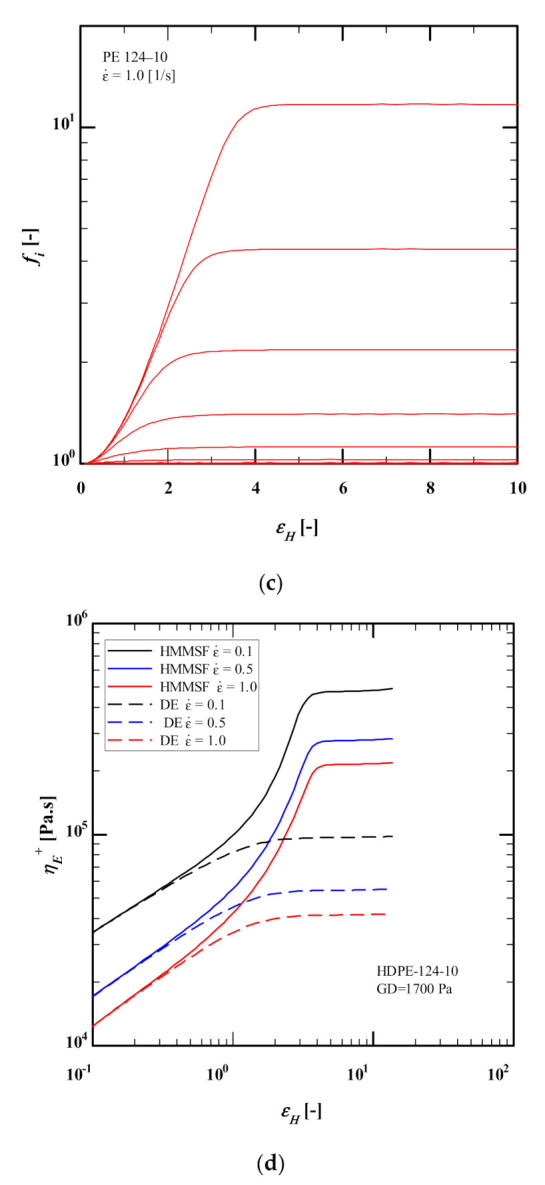
Spectral decomposition of stretch as a function of Hencky strain for PE 124-10 at (**a**) $\dot{\varepsilon }=0.1/s$, (**b**) $\dot{\varepsilon }=0.5/s$, (**c**) $\dot{\varepsilon }=1/s$ and (**d**) comparison between the predictions of the DE and HMMSF models as functions of Hencky strain.

To further investigate the stretch saturation observed in Figure 10, the relaxation spectral dependence of the stretch *f* (obtained from evolution equation Equation (6)) for PE124-10 at $\dot{\varepsilon }=0.1,\;0.5,\;1/s$ is plotted in Figure 11. The spectral dependence demonstrates the importance of the modes with longest relaxation times $\tau_{i}$, which feature the high dynamic dilution and, subsequently, lower mass fraction $w_{i}$ after dilution; hence, these modes have a large effect on the prediction of the elongational viscosity [25].

It is evident (Figure 11a–c) that the stretch is fully saturated at Hencky strain $\varepsilon_{H}=5$ for all strain rates. Moreover, it explains the minor difference observed between the values measured by our proposed MSHF Equation (13) at $\dot{\varepsilon }=0.1\;,\;0.5,\;1/s$. As shown in Figure 11a–c, only the four largest modes show $f_{i}>1$ at $\dot{\varepsilon }=0.1\;/s$ while $f_{i}>1$ is seen in five largest modes at $\dot{\varepsilon }=0.5,\;1/s$.

Figure 11d compares the predictions of the DE and HMMSF models as functions of Hencky strain for PE 124-10 obtained from the IRIS software. The inverse relation between the steady-state viscosity and strain rate is evident by both HMMSF and DE models.

Overall, it can be concluded that our proposed MSHF method delivers rate-insensitive and LCB-dependent results which are in agreement with the sheer effect of branching on the extent of the strain hardening behavior.

**Figure 12:**
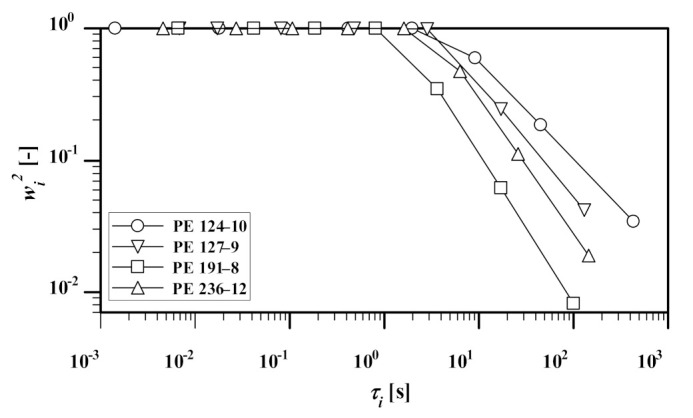
Mass fraction of dynamically diluted chains $w_{i}^{2}$ as a function of polydispersity and relaxation modes.

Figure 12 illustrates the mass fraction of dynamically diluted chains $w_{i}^{2}$ (Equation (5) (see also figure 3 of [30]) as a function of relaxation modes and PDI of the samples. Results show that (except for PE 236-12) the polydispersity of the HDPE samples has a direct relation with their $w_{i}^{2}$; i.e., an increase in PDI leads to the growth of the mass fraction. In other words, polydispersity enhances the dynamic dilution through the broadening of the relaxation spectrum. The same trend was observed for disengagement time (or LCBI) as a function of PDI (Figure 6), and $G_{D}$ as a function of PDI (Figure 6).

## 5. Conclusions

In this study, we showed the successful application of the HMMSF model on the elongational data of four sets of high-density polyethylene (PE 191-8, PE 127-9, PE 124-10 and PE 236-12) with traces of LCB. The HMMSF model, with only one nonlinear material parameter in uniaxial and multiaxial extensional flows, namely the dilution modulus $G_{D}$, shows excellent prediction of rheological properties of the tested polymers.

We propose a new method for investigating the strength of the strain hardening behavior in polymers based on molecular modelling by determining the ratio between the steady-state viscosities of the HMMSF model ($\eta_{E}(\dot{\varepsilon })$) and the DE model ($\eta_{DE}(\dot{\varepsilon })$) at a Hencky strain well within the steady-state regime.

The polydispersity of the samples has a direct relation with the mass fraction of dynamically diluted chains, $w_{i}^{2}$, i.e., an increase in PDI leads to the growth of the mass fraction. In other words, polydispersity enhances the dynamic dilution through broadening the relaxation spectrum.

## Figures and Tables

**Table 1:** Discrete relaxation spectrum and zero-shear viscosity of PE 191-8, PE 127-9, PE 124-10 and PE 236-12 (at T = 160 °C) by us using the IRIS software (green lines), and by Morelly and Alvarez [23], respectively.

PE 191-8 (IRIS).		PE 127-9 (IRIS)		PE 124-10 (IRIS)		PE 236-12 (IRIS)	
$g_{i}\;[\mathrm{Pa}]$	$\tau_{i}\;[s]$	$g_{i}\;[\mathrm{Pa}]$	$\tau_{i}\;[s]$	$g_{i}\;[\mathrm{Pa}]$	$\tau_{i}\;[s]$	$g_{i}\;[\mathrm{Pa}]$	$\tau_{i}\;[s]$
3.161 × 10^5^	6.605 × 10^−3^	3.435 × 10^4^	1.724 × 10^−2^	3.593 × 10^5^	1.432 × 10^−3^	2.895 × 10^5^	4.577 × 10^−3^
6.551 × 10^4^	4.203 × 10^−2^	1.382 × 10^5^	6.802 × 10^−3^	5.105 × 10^4^	1.783 × 10^−2^	6.273 × 10^4^	2.720 × 10^−2^
2.153 × 10^4^	1.856 × 10^−1^	2.855 × 10^4^	8.031 × 10^−2^	2.203 × 10^4^	8.784 × 10^−2^	2.630 × 10^4^	1.060 × 10^−1^
5.755 × 10^3^	8.070 × 10^−1^	9.711 × 10^3^	4.811 × 10^−1^	9.028 × 10^3^	4.148 × 10^−1^	9.387 × 10^3^	4.105 × 10^−1^
1.274 × 10^3^	3.638 × 10^0^	2.932 × 10^3^	2.859 × 10^0^	3.559 × 10^3^	1.953 × 10^0^	2.944 × 10^3^	1.616 × 10^0^
2.290 × 10^2^	1.695 × 10^1^	7.638 × 10^2^	1.719 × 10^1^	1.258 × 10^3^	9.099 × 10^0^	8.109 × 10^2^	6.347 × 10^0^
4.692 × 10^1^	9.925 × 10^1^	2.257 × 10^2^	1.288 × 10^2^	4.431 × 10^2^	4.527 × 10^1^	1.946 × 10^2^	2.611 × 10^1^
-	-	-	-	1.581 × 10^2^	4.298 × 10^2^	5.934 × 10^1^	1.449 × 10^2^
*η*_0@160°C_ = 26,650 Pas		*η*_0@160°C_ = 59,090 Pas		*η*_0@160°C_ = 113,500 Pas		*η*_0@160°C_ = 33,250 Pas	

## Data Availability

Data are available from the authors on request.
